# Transgenic *Tmc2* expression preserves inner ear hair cells and vestibular function in mice lacking *Tmc1*

**DOI:** 10.1038/s41598-018-28958-x

**Published:** 2018-08-14

**Authors:** Yukako Asai, Bifeng Pan, Carl Nist-Lund, Alice Galvin, Andrei N. Lukashkin, Victoria A. Lukashkina, Tianwen Chen, Wu Zhou, Hong Zhu, Ian J. Russell, Jeffrey R. Holt, Gwenaelle S. G. Géléoc

**Affiliations:** 1 Department of Otolaryngology and Communication Enhancement, F.M. Kirby Center for Neurobiology, Boston Children’s Hospital, Harvard Medical School, Boston, MA USA; 2 Sensory Neuroscience Research Group, School of Pharmacy and Biomolecular Sciences, University of Brighton, Brighton, UK; 3 Department of Otolaryngology and Communicative Sciences, University of Mississippi Medical Center, Oxford, MS USA; 4 Department of Neurology, Boston Children’s Hospital, Harvard Medical School, Boston, MA USA

## Abstract

Recent work has demonstrated that transmembrane channel-like 1 protein (TMC1) is an essential component of the sensory transduction complex in hair cells of the inner ear. A closely related homolog, TMC2, is expressed transiently in the neonatal mouse cochlea and can enable sensory transduction in *Tmc1*-null mice during the first postnatal week. Both TMC1 and TMC2 are expressed at adult stages in mouse vestibular hair cells. The extent to which TMC1 and TMC2 can substitute for each other is unknown. Several biophysical differences between TMC1 and TMC2 suggest these proteins perform similar but not identical functions. To investigate these differences, and whether TMC2 can substitute for TMC1 in mature hair cells, we generated a knock-in mouse model allowing *Cre*-inducible expression of *Tmc2*. We assayed for changes in hair cell sensory transduction and auditory and vestibular function in *Tmc2* knockin mice (*Tm*[*Tmc2*]) in the presence or absence of endogenous *Tmc1*, *Tmc2* or both. Our results show that expression of Tm[TMC2] restores sensory transduction in vestibular hair cells and transiently in cochlear hair cells in the absence of TMC1. The cellular rescue leads to recovery of balance but not auditory function. We conclude that TMC1 provides some additional necessary function, not provided by TMC2.

## Introduction

Sensory hair cells of the inner ear convert mechanical signals into electrical signals by means of a sensory transduction complex^1,2^ that resides at the tips of stereocilia^3^. While several components of this complex have now been identified, the molecular composition of the mechanosensory channel remains unclear, but is believed to include transmembrane channel-like proteins (TMC). *Tmc* genes were identified 15 years ago through positional cloning of a gene, *TMC1*, underlying both dominant and recessive nonsyndromic sensorineural hearing loss^4,5^. Mouse *Tmc1*, and its closely related paralog *Tmc2*, are expressed in the developing ear. In the auditory epithelium, initial tonotopic (base to apex) and transient expression of *Tmc2* is followed by sustained *Tmc1* expression beginning after postnatal day three (P3)^5^. The rise of *Tmc2* expression in the auditory organs coincides with the developmental acquisition of sensory transduction in cochlear hair cells. The tonotopic decrease in *Tmc2* expression occurs during the first and second postnatal week^6,7^. In the vestibular system, *Tmc1* and *Tmc2* expression rises during development, and, in contrast with the cochlea, *Tmc2* expression is maintained in mature hair cells. Sensory transduction is maintained throughout the utricle in absence of *Tmc1* and is restricted to the extra-striolar region in absence of *Tmc2*. Mice lacking both *Tmc1* and *Tmc2* lack sensory transduction currents in auditory or vestibular hair cell from all regions and time points examined^7,8^. However, analysis of sensory transduction currents in hair cells of mice lacking only *Tmc1* or only *Tmc2* revealed nearly normal responses during the first postnatal week^7,8^. Interestingly, mice that lack *Tmc1* and *Tmc2* exhibit abnormal vestibular behavior, but mice that express *Tmc1* or *Tmc2* retain vestibular function^7^. Based on these observations, we hypothesize that TMC1 and TMC2 proteins have redundant functions, and that expression of either TMC1 or TMC2 may be sufficient for sensory transduction, auditory and vestibular function. To explore the temporal requirements of *Tmc2* gene expression and the functional redundancy between *Tmc1* and *Tmc2*, we generated a targeted knock-in mouse model allowing *Cre*-inducible expression of *Tmc2*. We assayed for changes in hair cell sensory transduction and auditory and vestibular function in the developing and mature *Cre*-recombined targeted knock-in *Tmc2* mice (referred to as *Tm*[*Tmc2*]) in presence or absence of endogenous *Tmc1*, *Tmc2*, or both. We show that cochlear hair cells from mice lacking *Tmc1* but expressing *Tm*[*Tmc2*], acquire sensory transduction at earlier stages and maintain sensory transduction for an extended period relative to *Tmc1*-null hair cells. High threshold auditory brainstem responses were observed initially in the *Tm*[*Tmc2*] mice but were absent after 6 weeks of age. Our results show that *Tm*[*Tmc2*] can partially compensate for *Tmc1* but cannot substitute long-term for the absence of *Tmc1* in the cochlea. In the vestibular system, *Tm*[*Tmc2*] mice maintained normal sensory transduction in hair cells and expression of the *Tm*[*Tmc2*] gene in the absence of endogenous *Tmc1* and *Tmc2* preserved normal balance behavior, which is otherwise compromised in double *Tmc1/Tmc2*-null mice.

## Results

### Generation of *Rosa26*^*tm*^*(CAG-LSL-Tmc2-IRES-GFP)* mouse model (*Tm[Tmc2]*)

**Figure 1:**
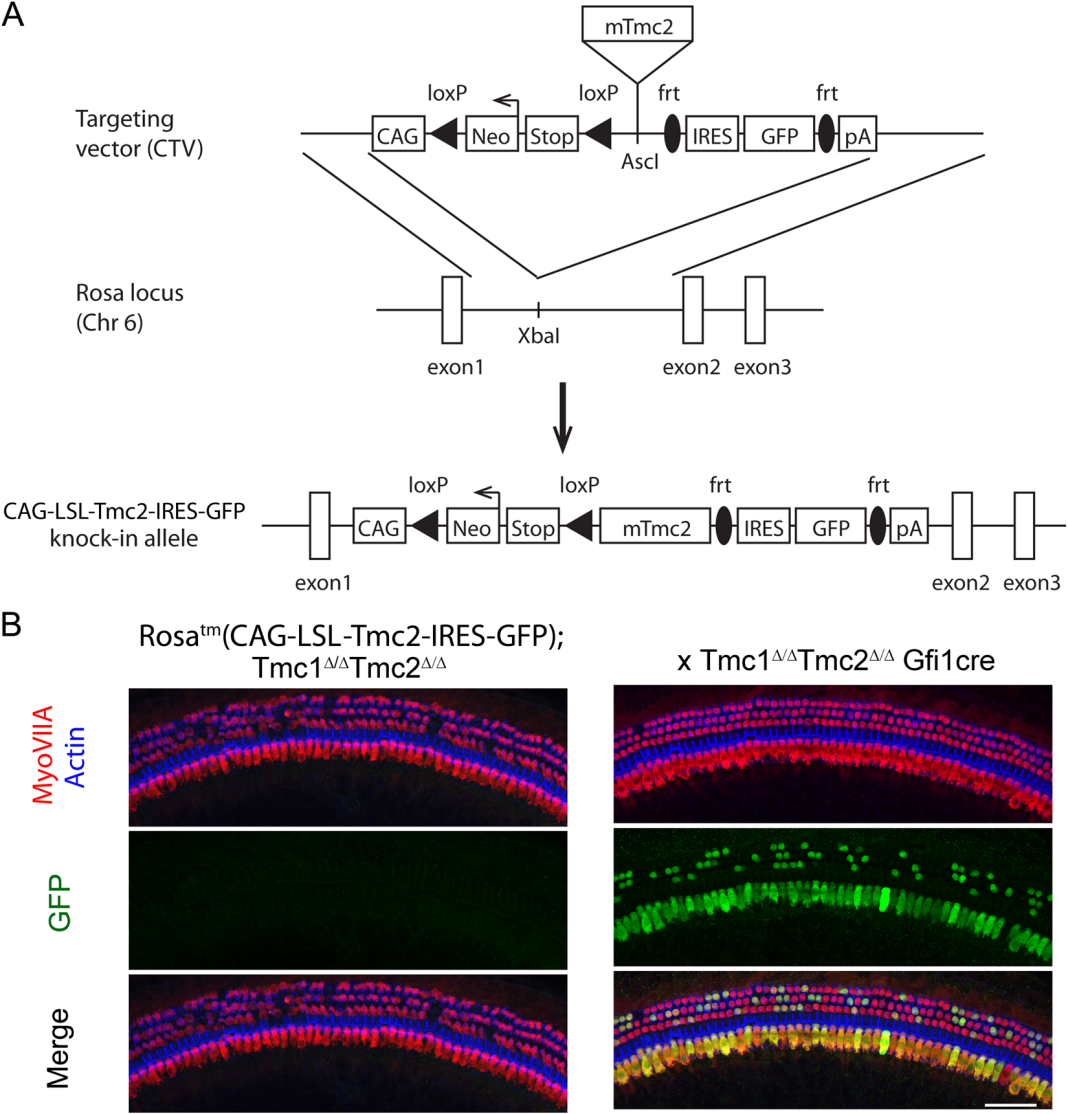
Generation of *Rosa26*^*tm*^(*CAG-LSL-Tmc2-IRES-GFP*) mouse model. (**A**) Mouse *Tmc2* cDNA was inserted into the *CAG-STOP-GFP-Rosa* targeting vector, CTV, between a floxed Stop cassette and the internal ribosome entry site (IRES) followed by the enhanced Green Fluorescent Protein gene (eGFP). Transcription is under control of the CAG promoter. The targeting vector contained *Rosa26* homology arms (1 kb 5′ and 3.8 kb 3′), so that the entire *loxP-stop-loxP-Tmc2-IRES-GFP* transcriptional cassette was inserted into the first intron of *Rosa26* gene on chromosome 6. (**B**) When expressed with *Gfi1*^*Cre*^, excision of the stop codon lead to expression of *Tm*[*Tmc2*] and GFP in IHCs and OHCs. GFP expression was evident at P16. Hair cells were labeled with rabbit anti-MYOVIIa (red), GFP signal is amplified with a goat anti-GFP antibody (green) and phalloidin counterstain labeled actin filaments (blue). Scale bar 50 μm.

The ability of *Tmc2* to substitute for *Tmc1* was investigated using *Cre-loxP* recombination to conditionally express a knocked-in *Tmc2* gene (referred to as *Tm*[*Tmc2*]) in hair cells. We generated a mouse line, the *Rosa26*^*tm*^(*CAG-LSL-Tmc2-IRES-GFP*), by knocking into the *Rosa26* locus, a CAG promoter driving loxP-flanked stop codons (*loxP-stop-loxP or LSL*) upstream of *Tm*[*Tmc2*] coding sequences, along with the sequence for a green fluorescent protein (GFP) reporter downstream of an IRES sequence (Fig. 1A). Constitutive expression of *Tm*[*Tmc2*] was obtained by crossing *Rosa26*^*tm*^(*CAG-LSL-Tmc2-IRES-GFP*) mice with inner ear specific *Cre*-expressing mice. Both the *Rosa26*^*tm*^(*CAG-LSL-Tmc2-IRES-GFP*) and Cre mice (*Gfi1*^*Cre*,^ ^9^ or *VT3*^*Cre*,^ ^10^) were crossed to *Tmc1/Tmc2*-null mice to obtain different genotypes: *Tmc1*^*Δ/Δ*^, *Tmc2*^*Δ/Δ*^, or *Tmc1*^*Δ/Δ*^*Tmc2*^*Δ/Δ*^ double knockouts. Expression of the knockin construct was assessed by observing GFP expression in inner ear organs. No GFP expression was detected in the *Rosa26*^*tm*^(*CAG-LSL-Tmc2-IRES-GFP*) mice (Fig. 1B). When mice were crossed with *Gfi1*^*Cre*^ mice, recombination was observed in all IHCs and a mosaic GFP pattern was seen in OHCs both at P16 and 8 weeks (Fig. 1B). GFP expression was restricted to sensory hair cells.

**Figure 2:**
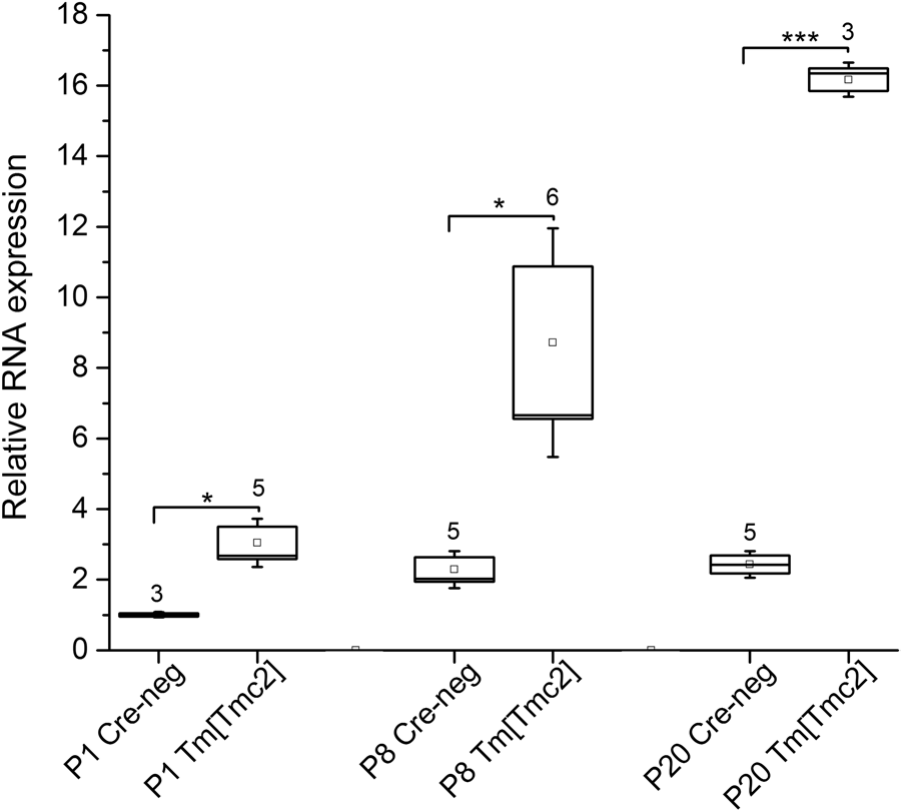
Relative expression of GFP in *Rosa26*^*tm*^(*CAG-LSL-Tmc2-IRES-GFP*) *mice*. Total RNA was prepared from single cochlea harvested from *Rosa26*^*tm*^(*CAG-LSL-Tmc2-IRES-GFP*)]*;Tmc1*^*Δ/Δ*^*Tmc2*^*Δ/*+^ positive for *Gfi1*^*cre*^ (*Tm*[*Tmc2*]) or negative for *Gfi1*^*cre*^ (*Cre-neg*) at P1, P8 and P20. Quantitative RT-PCR was performed with β-actin and GFP primers in triplicate (see methods) and repeated from biological replicates (number of samples are noted above the box plots). Due to bicistronic expression of *Tm*[*Tmc2*] and GFP in *Rosa26*^*tm*^(*CAG-LSL-Tmc2-IRES-GFP*) mice, the expression level of GFP reflected *Tm*[*Tmc2*] expression. The cycle threshold (Ct) for each sample was normalized relative to the Ct value of β actin from the same sample. Relative expression was calculated by normalization to P1 *Gfi1*^*Cre*^ negative control samples. Significant expression of the knockin gene was detected at each stages from P1 to P20. Data are represented in box plots as means ± S.E.M. Statistical analysis were performed using the independent t-test. *p < 0.05; **p < 0.01; ***p < 0.001.

To assess expression of *Tm*[*Tmc2*] from the *Rosa26* locus, quantitative RT-PCR was performed using primers selective for *GFP. GFP* expression is correlated to that of *Tm*[*Tmc2*] because mRNA encoding for both genes is generated as a single bicistronic transcript (Fig. 1A). Total RNA was prepared from *Rosa26*^*tm*^(*CAG-LSL-Tmc2-IRES-GFP*) mice under *Tmc1*^*Δ/Δ*^*Tmc2*^*Δ/*+^ background crossed to *Gfi1*^*Cre*^ expressing mice at P1, P8 and P20. The relative difference in *GFP* mRNA expression in presence of *Gfi1*^*Cre*^ was 3-fold at P1, 5.6-fold at P8 and 6.6-fold at P20 (Fig. 2).

For simplicity, we refer to mice with *Gfi1*^*cre*^ recombined with *Rosa26*^*tm*^(*CAG-LSL-Tmc2-IRES-GFP*) knockin mice as *Tm*[*Tmc2*] mice in the rest of the manuscript. When knockin mice have been recombined to *VT3*^*Cre*^, they are referred to as *Tm*[*Tmc2*]^*VT3-cre*^.

### Expression of Tm[TMC2] protein shifts acquisition of sensory transduction earlier

**Figure 3:**
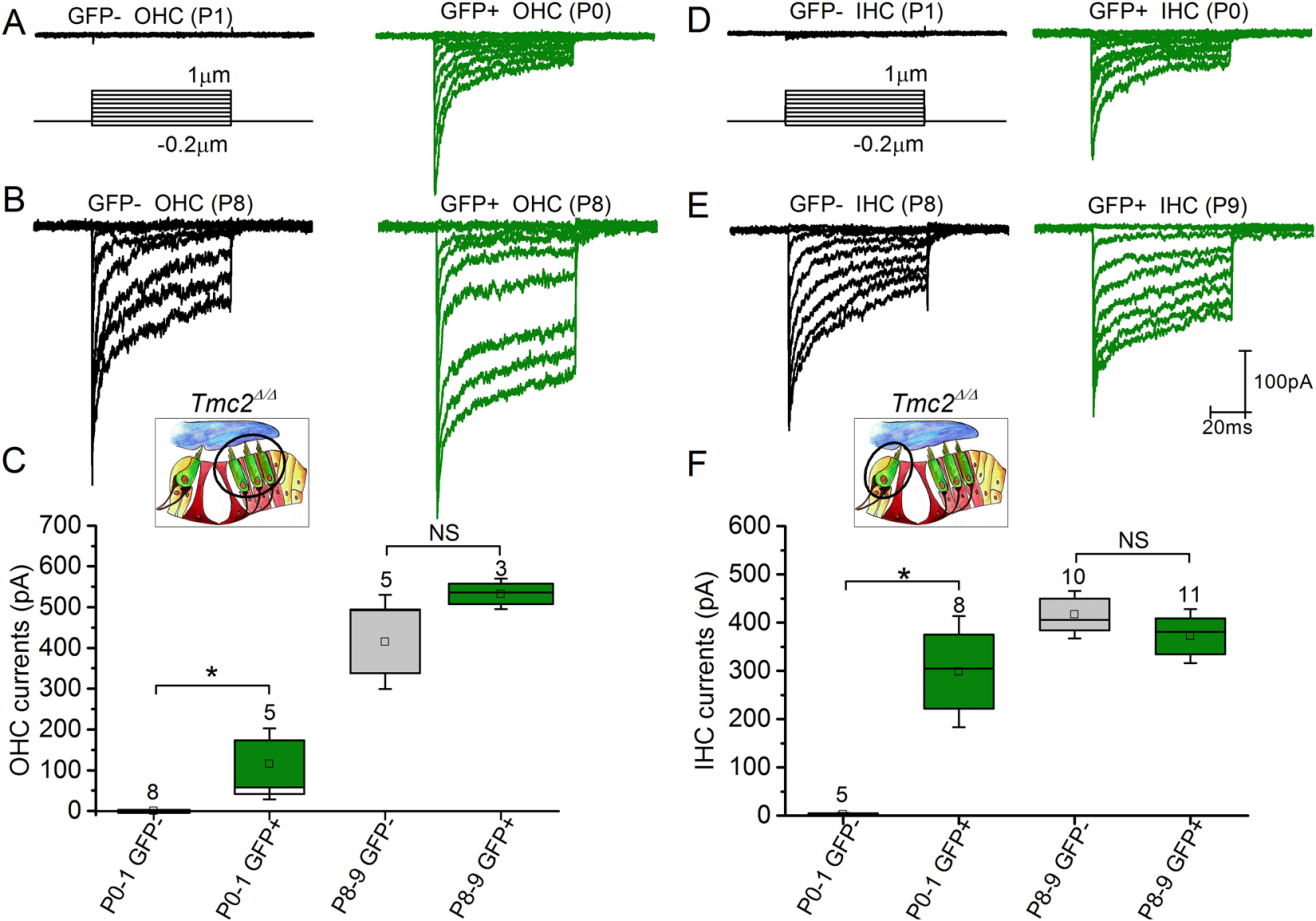
Sensory transduction currents in *Rosa26*^*tm*^(*CAG-LSL-Tmc2-IRES-GFP*);*Tmc2*^*Δ/Δ*^ mice. (**A**–**C**) Sensory transduction currents were recorded from P0-P8 GFP-positive and GFP-negative OHCs of *Rosa26*^*tm*^(*CAG-LSL-Tmc2-IRES-GFP*)*;Gfi1*^*Cre/*+^*;Tmc2*^*Δ/Δ*^ mice. While absent in GFP-negative P0 apical OHCs, large currents were recorded in P0-P1 apical GFP-positive cells. Non-significant difference was noted by P8. (**D,E**) Sensory transduction currents were recorded from P0-P9 GFP-positive and GFP-negative IHCs. While absent in GFP- negative P0 apical OHCs, large currents were recorded in P0-P1 apical GFP-positive cells. There was no significant difference at P8-P9. Number of cells is indicated for each group. Mean ± S.E.M. **p < 0.01; NS p > 0.05 (one way ANOVA).

To evaluate the consequences of Tm[TMC2] expression in the absence of endogenous TMC2 protein, we obtained electrophysiological recordings from hair cells of *Tm*[*Tmc2*]*; Tmc2*^*Δ/Δ*^ mice. Hair bundle deflections were evoked using stiff glass probes shaped to fit the bundles of inner and outer hair cell stereocilia. The pipettes were mounted on a stack of piezoelectric actuators that enabled rapid deflections, as previously described^11^. Since *Cre* expression in the inner ear of *Gfi1*^*Cre/*+^ coincides with hair cell formation^9^, we wondered if GFP-positive cells could be observed at P0. Indeed, we observed the presence of GFP-positive cells and adjacent GFP-negative cells at P0-P1. Transduction currents were recorded from both GFP-positive and GFP-negative cells (Fig. 3) in 1.3 mM external calcium at a holding potential of −84 mV. Robust sensory transduction currents were present at postnatal days P0-P1 in GFP-positive apical OHCs and IHCs (115.8 ± 58.0 pA, n = 5 OHCs; 298.4 ± 76.7 pA n = 8 IHCs) but absent in GFP-negative apical cells (n=8 OHCs, n = 5 IHCs) (Fig. 3A,C,D,F). The currents resembled those of mature hair cells, albeit with smaller amplitudes (Fig. 3C,F). Adaptation was always present, with fast and slow time constants of 2.05 ± 0.37 ms, 20.00 ± 1.54 ms, respectively, and extent of adaptation at 76.5 ± 3.3% (n = 5) in P0-P1 GFP-positive OHCs and fast and slow time constants of 2.08 ± 0.49 ms, 22.8 ± 0.50 ms, respectively, and extent of adaptation at 76.4 ± 3.3% (n = 8) in P0-P1 GFP-positive IHCs. Time constants of adaptation were similar to what has been reported previously for wild type neonatal OHCs and IHCs^11,12^. By P8-P9, sensory transduction currents were detected in all cells and their amplitudes did not differ significantly (P > 0.3) between cells expressing *Tm*[*Tmc2*] (GFP-positive OHCs: 415 ± 77.1 pA, n = 5; IHCs: 416.8 ± 32.8 pA, n = 10) and those that did not (GFP-negative OHCs: 532.7 ± 24.9 pA, n = 3; IHCs: 372.0 ± 37.4 pA, n = 11) (Fig. 3B,C,E,F). The data demonstrate that *Tm*[*Tmc2*] expression leads to earlier acquisition of sensory transduction in both IHCs and OHCs in the absence of endogenous *Tmc2*.

### Expression of Tm[TMC2] protein results in larger sensory transduction currents in cochlear hair cells lacking TMC1

**Figure 4:**
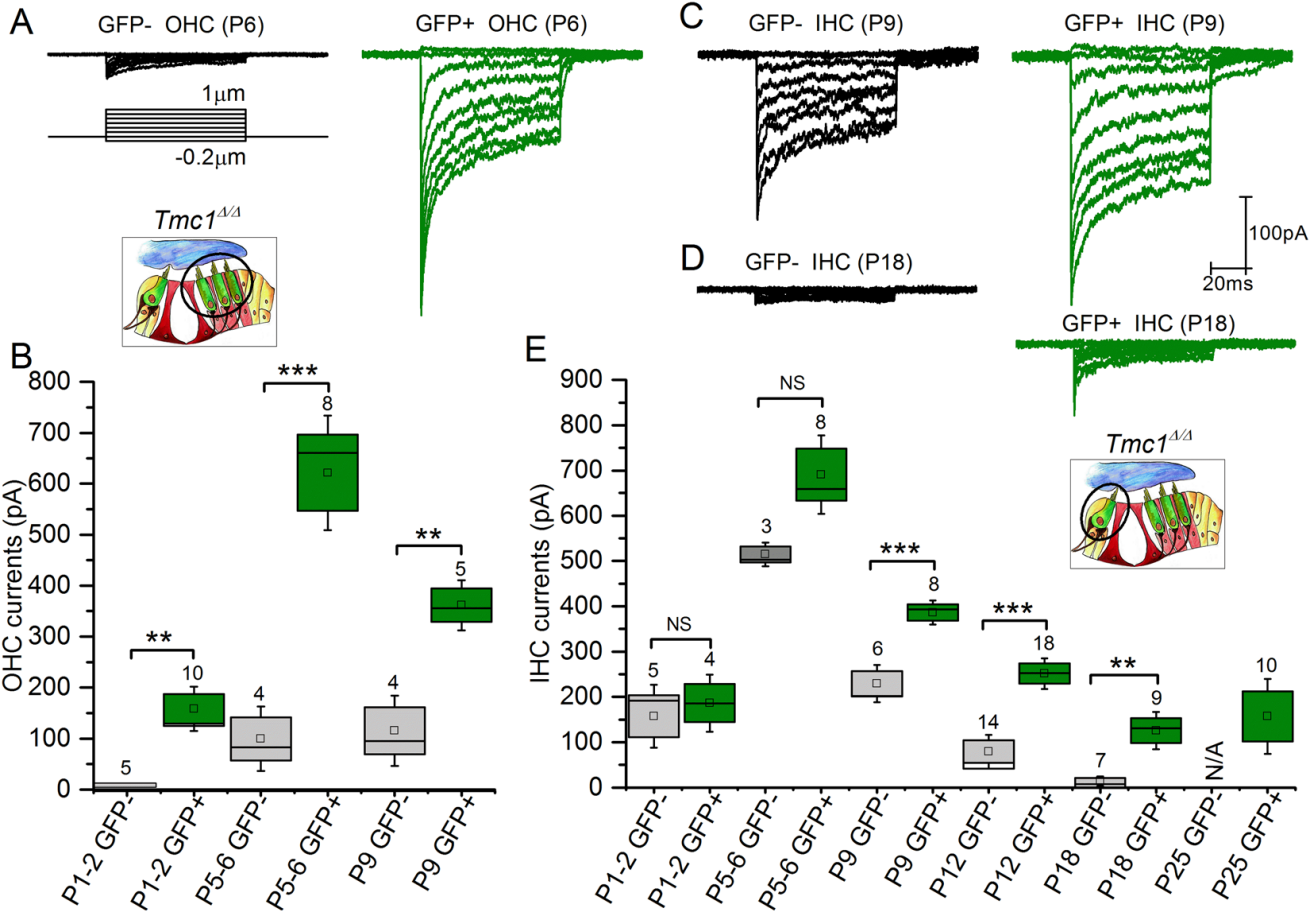
Sensory transduction in *Rosa26*^*tm*^(*CAG-LSL-Tmc2-IRES-GFP*);*Tmc1*^*Δ/Δ*^ mice. (**A**,**B**) Sensory transduction currents were recorded from P1-9 GFP-positive and GFP-negative OHCs of *Rosa26*^*tm*^(*CAG-LSL-Tmc2-IRES-GFP*)*;Gfi1*^*Cre/*+^*;Tmc1*^*Δ/Δ*^ mice. While absent in GFP-negative P1-P2 OHCs, large currents were recorded in P1-P2 GFP-positive cells. (**C**–**E)** Currents were recorded from P1-P25 GFP-positive and P1-P18 GFP-negative IHCs. While currents decreased rapidly in amplitude after P6, large currents were measured GFP-positive cells as late as P25. Number of cells is indicated above the bar graphs. Mean ± S.E.M. ***p < 0.001; **p < 0.01; NS p > 0.05 (one way ANOVA). N/A, non-applicable: GFP-negative IHCs were absent at older stages.

To evaluate the consequences of Tm[TMC2] expression in the absence of endogenous TMC1 protein, we obtained electrophysiological recordings from hair cells of *Tm*[*Tmc2*]*;Tmc1*^*Δ/Δ*^ mice at a holding potential of −84mV. Stable whole cell recordings could not be obtained after P12 in OHCs. We therefore recorded transduction currents in GFP-positive and GFP-negative OHCs between P1 and P9, and IHCS between P1 and P25 (Fig. 4). While currents were not detected in GFP-negative apical OHCs of P1- P2 mice, small currents were measured in GFP-positive cells that expressed *Tm*[*Tmc2*] (158.0 ± 29.0 pA, n = 10; Fig. 4B) suggesting that *Tm*[*Tmc2*] was expressed earlier than its endogenous counterpart. By P5-P6, significantly larger transduction currents were present in GFP-positive OHCs in comparison to GFP-negative OHCs (P > 0.001; Fig. 4A,B). Similar currents were recorded in neonatal P1-P6 IHCs in presence or absence of the *Tm*[*Tmc2*] (Fig. 4E). While IHCs transduction currents declined rapidly after P6 in GFP-negative cells, they were maintained in GFP-positive cells (Fig. 4D,E): at P25, small but steady currents were measure in GFP-positive IHCs (157.1 ± 55.0 pA, n = 10; Fig. 4E). These results demonstrate that expression of *Tm*[*Tmc2*] in the absence of *Tmc1* preserves IHCs and sensory transduction up to P25 (the latest stage tested).

### Expression of Tm[TMC2] protein restores sensory transduction in *Tmc1*/*Tmc2*-double knockout mice

**Figure 5:**
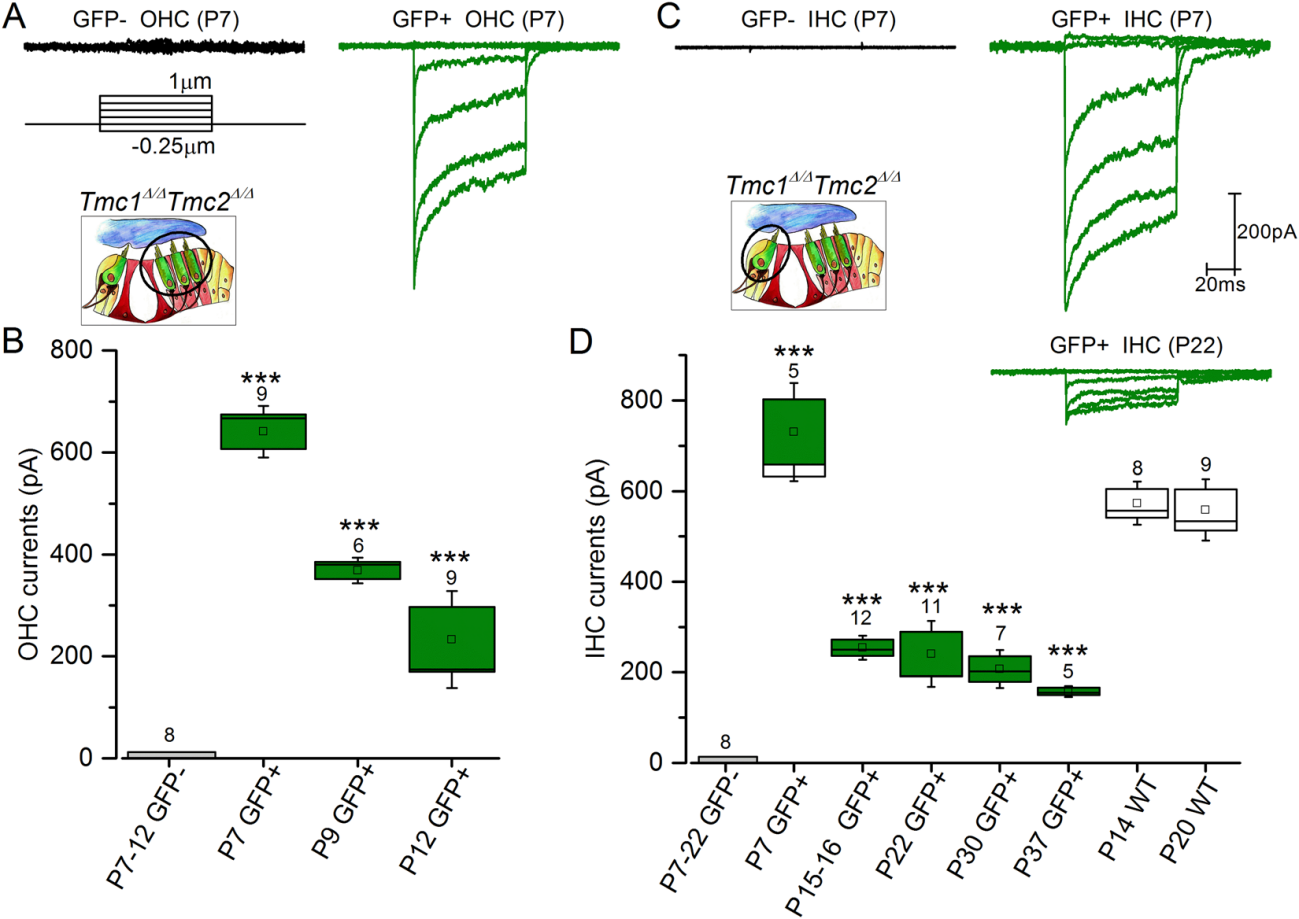
Sensory transduction in *Rosa26*^*tm*^(*CAG-LSL-Tmc2-IRES-GFP*)/*Tmc1*^*Δ/Δ*^*Tmc2*^*Δ/Δ*^ mice. (**A**,**B**) Sensory transduction currents were recorded from P7-P12 GFP-positive and GFP-negative OHCs. While absent in GFP-negative OHCs at all stages recorded, large currents were recorded in P7-P12 GFP-positive cells. (**C**,**D)** Currents were recorded from P7-P37 GFP- positive and P7-P22 GFP-negative IHCs. Large currents were measured in P7 GFP-positive cells. Currents were maintained albeit reduced after P15. In comparison, sensory transduction currents were larger and stable in IHCs of wild type mice at P14 and P20. Number of cells is indicated. Mean ± S.E.M. ***p < 0.001 (one way ANOVA).

To fully assess the extent of Tm[TMC2] functional compensation in hair cells, we performed similar recordings in cells devoid of endogenous TMC1 and TMC2 proteins. Sensory transduction currents were recorded in OHCs and IHCs of *Tm*[*Tmc2*]*;Tmc1*^*Δ/Δ*^*;Tmc2*^*Δ/Δ*^ mice (Fig. 5). GFP-negative cells, which lack Tm[TMC2] and endogenous TMC1 and TMC2 proteins, are predicted to lack sensory transduction entirely. Our data confirm that this was indeed the case as none of the GFP-negative cells responded to hair bundle deflections (n = 8 OHCs and n = 8 IHCs, P7-P22; Fig. 5A–D). In contrast, large sensory transduction currents were present at P7 in GFP-positive OHCs and were maintained up to P12, the latest stage at which reliable OHCs recordings were obtained (Fig. 5B). Similarly, large currents were recorded in P7 GFP-positive IHCs. Currents declined after P7 but significant transduction currents (>100pA) remained in GFP-positive cells up to P37, the latest stage tested (Fig. 5D). In contrast, large sensory transduction currents were elicited from IHCs of wild type mice which remained stable from P14 to P20. These results therefore demonstrate that *Tm*[*Tmc2*] can partially compensate for the absence of endogenous*Tmc1* and *Tmc2* in cochlear hair cells. At P7, adaptation in *Tm*[*Tmc2*]*;Tmc1*^*Δ/Δ*^*;Tmc2*^*Δ/Δ*^ OHCs was similar to that of P0-P1 neonatal OHCs. In comparison, slower adaptation was observed in IHCs of P7 *Tm*[*Tmc2*]*;Tmc1*^*Δ/Δ*^*;Tmc2*^*Δ/Δ*^ mice. Fast and slow time constants at P7 were 1.87 ± 1.24 ms, 24.76 ± 7.83 ms, respectively, and the extent of adaptation was 80.0 ± 9.1% (n = 9) in GFP positive OHCs. In GFP-positive IHCs adaptation time constants were 4.48 ± 2.36 ms (fast), 34.64 ± 15.13 ms (slow) with an extent of adaptation of 62.4 ± 7.0% (n = 5). These values are similar to what has been reported previously for hair cells of wild type mice^11^.

### Tm[TMC2] protein expression transiently restores auditory brainstem responses

**Figure 6:**
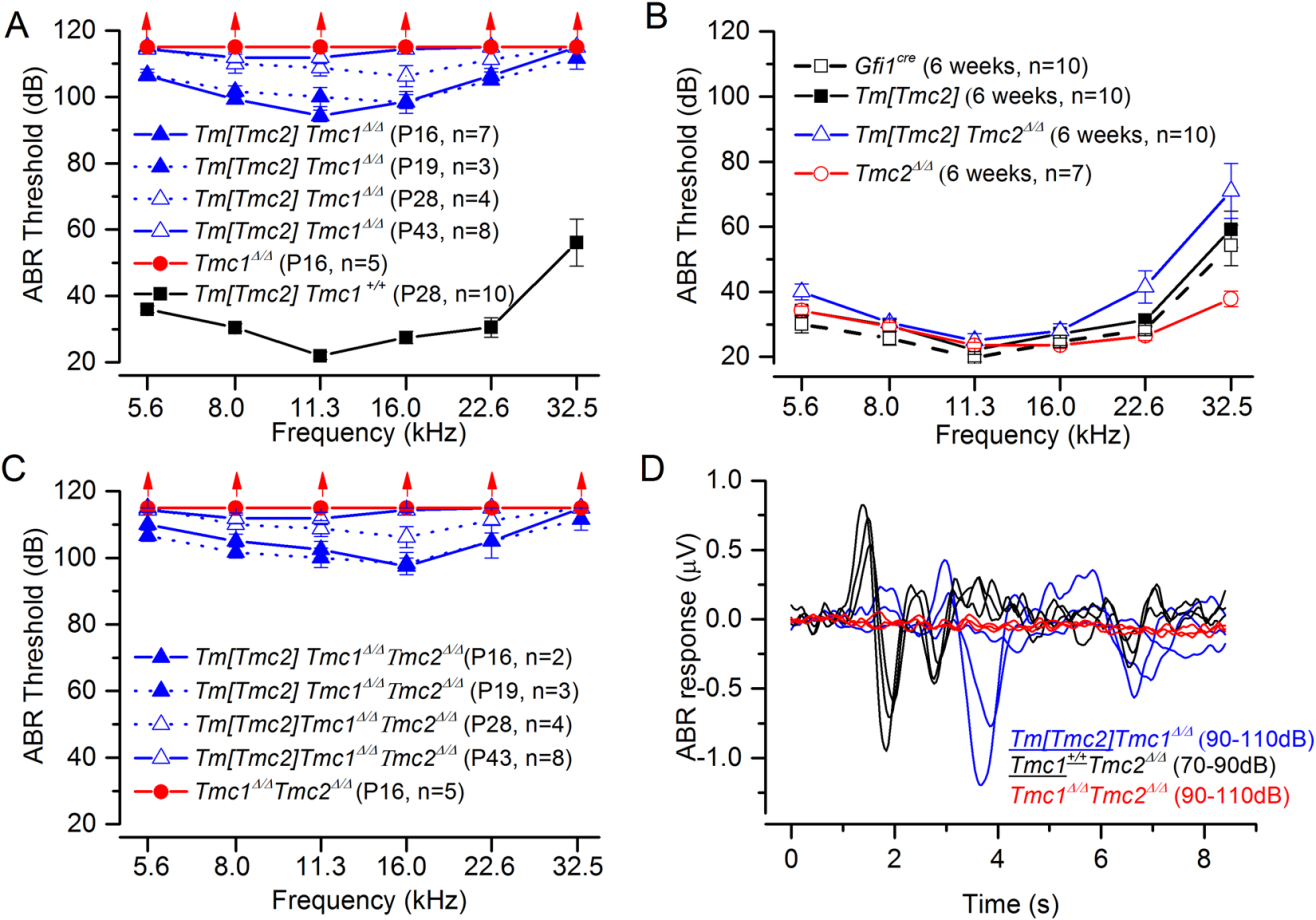
*Gfi1*^Cre^ inducible expression of *Tm*[*Tmc2*] in absence of Tmc1 restore mild ABR sensitivity. Auditory brainstem responses were recorded in knockin mice crossed to *Gfi1*^*Cre*^ expressing mice to obtain *Tm*[*Tmc2*] expression in absence of *Tmc1* (**A**), *Tmc2* (**B**) or both (**C**). ABR were detected for sound levels above 80 dB in mice that expressed the *Tm*[*Tmc2*] in absence of *Tmc1* or *Tmc1* and *Tmc2* at P16. Responses gradually disappeared and the mice were profoundly deaf by 6 weeks of age (**A**,**C**). While mild high frequency hearing loss was observed in mice expressing *Tm*[*Tmc2*] in presence of *Tmc1*, the loss was also evident in *Gfi1*^*Cre*^ control mice (*Tmc1*^+*/*+^*Tmc2*^+*/*+^*Gfi1*^*cre/*+^) demonstrating that this feature resulted from background strains rather than expression of *Tm*[*Tmc2*] itself (**B**). Responses observed at P16 had longer latencies which resulted in altered ABR (**D**). Mean ± S.E.M.

To assess the ability of Tm[TMC2] protein to restore auditory function at the systems level, we recorded auditory brainstem responses (ABR) in *Tm*[*Tmc2*]*;Tmc1*^*Δ/Δ*^, *Tm*[*Tmc2*]*;Tmc2*^*Δ/Δ*^ mice, and *Tm*[*Tmc2*]*;Tmc1*^*Δ/Δ*^*;Tmc2*^*Δ/Δ*^. ABRs were detected for sound levels above 80 dB in mice that expressed *Tm*[*Tmc2*] in absence of TMC1 or both TMC1 and TMC2 at P16 (Fig. 6). Responses gradually diminished at later stages so that mice were profoundly deaf by 6 weeks of age (Fig. 6A,C). Although high frequency hearing loss was detected in 6 week-old mice expressing *Tm*[*Tmc2*] in presence of endogenous *Tmc1*, similar thresholds were observed in *Gfi1*^*Cre/*+^ control mice (Fig. 6B) suggesting that the *Gfi1*^*Cre*^ background accounted for the high frequency hearing loss^13^. Analysis of ABRs in mice expressing Tm[TMC2] showed that ABR waveforms had longer latencies at P16 (Fig. 6D).

**Figure 7:**
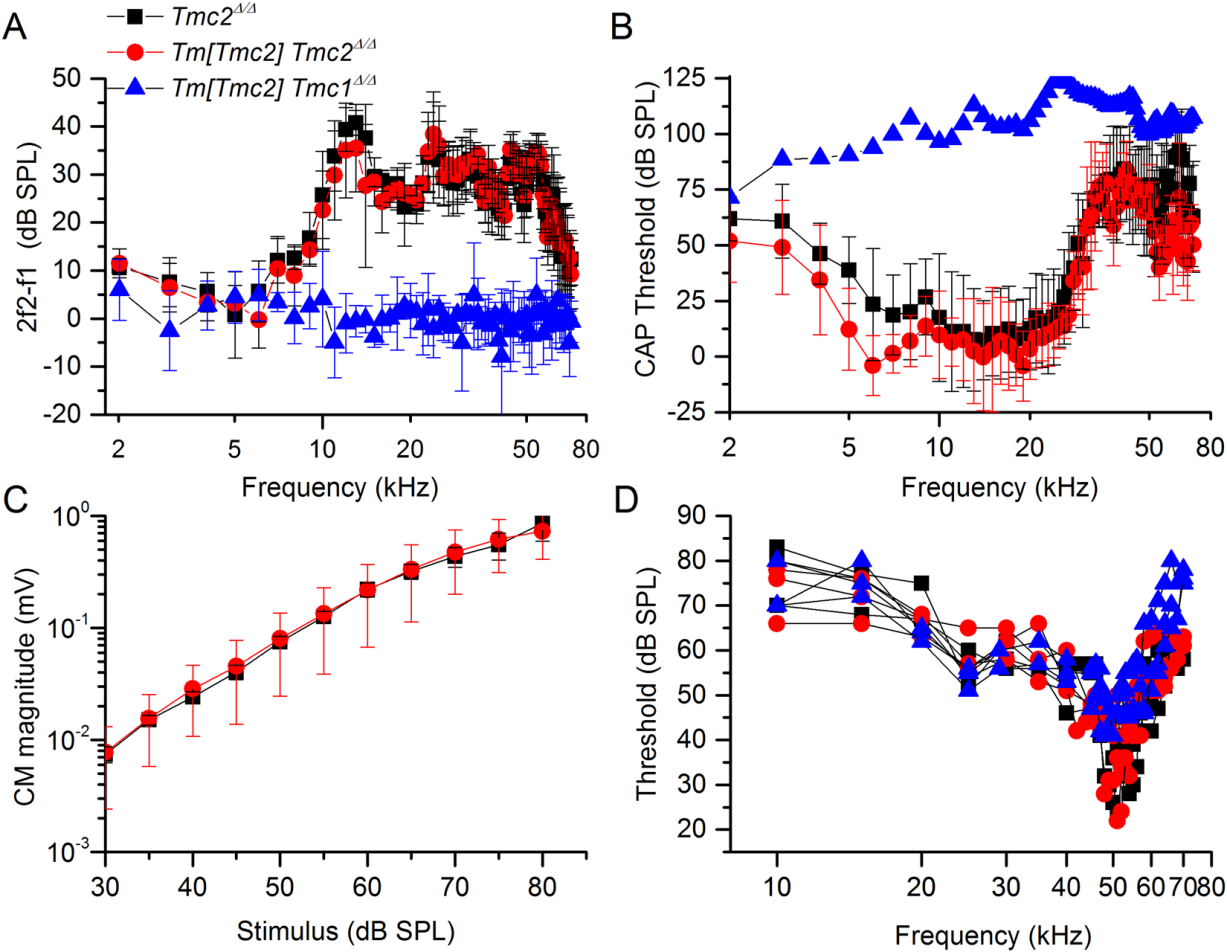
*Tm*[*Tmc2*] expression does not disrupt auditory function in *Tmc2*^*Δ/Δ*^ mice but fails to rescue auditory function in *Tmc1*^*Δ/Δ*^ mice. Recordings were performed from 8 *Tm*[*Tmc2*]*;Tmc2*^*Δ/Δ*^ (red), 7 *Tmc2*^*Δ/Δ*^ (without *Tm*[*Tmc2*], black) littermates, and 8 *Tm*[*Tmc2*]*;Tmc1*^*Δ/Δ*^ mice (blue). (**A**) Similar, sensitive, distortion product otoacoustic emission (DPOAE) audiograms (2f1-f2 DPOAE magnitude as function of f2 frequency, mean ± S.D.) recorded from *Tm*[*Tmc2*]*;Tmc2*^*Δ/Δ*^ *and Tmc2*^*Δ/Δ*^ mice. No DPOAEs were recorded above the noise floor (0 dB SPL) from *Tm*[*Tmc2*]*;Tmc1*^*Δ/Δ*^ mice. DPOAEs were recorded from the auditory meatus. F1 was set at 70 dB SPL and f2 was set at 60 dB SPL. (**B**) Similar, sensitive, compound action potential (CAP recorded from the round window) audiograms (CAP threshold as a function of stimulus frequency, mean ± S.D.) were recorded from *Tm*[*Tmc2*]*;Tmc2*^*Δ/Δ*^ and *Tmc2*^*Δ/Δ*^, but not from *Tm*[*Tmc2*]*;Tmc1*^*Δ/Δ*^ mice as indicated by the blue line (upper SPL of sound system). (**C**) Cochlear microphonic potential (CM) as a function of the level of the 5 kHz stimulus tone (mean ± S.D.) recorded from the round window are almost identical in *Tm*[*Tmc2*]*;Tmc2*^*Δ/Δ*^ *and Tmc2*^*Δ/Δ*^ mice. CM was not elicited from *Tm*[*Tmc2*]*;Tmc1*^*Δ/Δ*^ mice for any level of the 5 kHz tones. (**D**) Threshold, mechanical frequency tuning curves, based on 0.2 nm displacements recorded from the basilar membrane in cochlear basal turns are almost identical in *Tm*[*Tmc2*]*;Tmc2*^*Δ/Δ*^ *and Tmc2*^*Δ/Δ*^ mice. Tuning curves measured from *Tm*[*Tmc2*]*;Tmc1*^*Δ/Δ*^ mice resemble post-mortem responses from *Tm*[*Tmc2*]*;Tmc2*^*Δ/Δ*^ *and Tmc2*^*Δ/Δ*^, mice. Measurements were confined to mice aged P20–28.

To evaluate auditory function in greater detail, we recorded distortion product otoacoustic emissions (DPOAEs) from the auditory meatus, cochlear microphonic (CM) potentials and compound action potentials (CAPs) measured directly from the round window, and basilar membrane (BM) displacements from the high frequency turn of the cochlea from *Tm*[*Tmc2*]*;Tmc2*^*Δ/Δ*^ (n = 8), *Tmc2*^*Δ/Δ*^ mice (n = 7) and *Tm*[*Tmc2*]*;Tmc1*^*Δ/Δ*^ mice (n = 8) (Fig. 7). In *Tmc2*^*Δ/Δ*^ and *Tm*[*Tmc2*]*;Tmc2*^*Δ/Δ*^ mice (littermate controls), there was no difference in sensitivity and frequency range (up to the 70 kHz upper limit of the recording system) in DPOAE audiograms relative to control, which indicated normal OHC function (Fig. 7A). CAP audiograms were unchanged, indicating normal IHC responses (Fig. 7B). CM responses to 5 and 10 kHz tones were also unchanged, indicating functional transduction apparatus (Fig. 7C). Furthermore, the sensitivity and frequency tuning of the BM responses (Fig. 7D) (Q_10 dB_ characteristic frequency / bandwidth 10 dB from the tip) of *Tm*[*Tmc2*]*;Tmc2*^*Δ/Δ*^ (Q_10 dB_ = 10.3 ± 0.6, threshold at tip 28 ± 4 dB SPL, n = 5) and *Tmc2*^*Δ/Δ*^ control mice (Q_10 dB_ = 9.8 ± 0.7, threshold at tip 27 ± 6 dB SPL, n = 4) were not significantly different. BM measurements revealed that amplification, sensitivity and the frequency tuning in the cochleae *Tm*[*Tmc2*]*;Tmc2*^*Δ/Δ*^ and *Tmc2*^*Δ/Δ*^ were similar demonstrating that *Tm*[*Tmc2*] expression did not alter function (Fig. 7D).

To assess the ability of *Tm*[*Tmc2*] to functionally compensate for the absence of *Tmc1*, similar recordings were performed from eight *Tm*[*Tmc2*];*Tmc1*^*Δ/Δ*^ mice. Recordings were made from mice aged between P19 and 28. No DPOAEs were observed in *Tm*[*Tmc2*]*;Tmc1*^*Δ/Δ*^ mice for f2 frequencies between 2 kHz and 70 kHz and levels up to 70 dB SPL (Fig. 7A). This result was taken to indicate that OHCs were not providing amplification to the cochlear partition at any point along the length of the cochlea. Likewise, 5 and 10 kHz tones, which produce the same CM responses in *Tm*[*Tmc2*]*;Tmc2*^*Δ/Δ*^ and *Tmc2*^*Δ/Δ*^ mice (Fig. 7C), failed to elicit CM from *Tm*[*Tmc2*]*;Tmc1*^*Δ/Δ*^ mice at levels up to 125 dB SPL. This finding indicated that OHCs in the basal high frequency turn of the cochlea of *Tm*[*Tmc2*]*;Tmc1*^*Δ/Δ*^ mice were not generating receptor potentials. We were also unable to evoke CAPs in *Tm*[*Tmc2*]*;Tmc1*^*Δ/Δ*^ mice at frequencies between 4 kHz and 30 kHz (≤125 dB SPL) and for frequencies between 32 kHz and 70 kHz (≤105 dB SPL), which indicated that IHCs were not signaling cochlear responses to the auditory nerve. Similarly, absence of active tuning of BM responses indicated that OHCs are not functional in *Tm*[*Tmc2*]*;Tmc1*^*Δ/Δ*^ mice (Fig. 7D).

### Tm[TMC2] protein expression in IHCs does not alter auditory function

The knock-in *Rosa26*^*tm*^(*CAG-LSL-Tmc2-IRES-GFP*) mice were crossed to a vesicular glutamate transporter 3 *Cre* expressing mouse (*Vglut3-ires-Cre* knock-in mice: *VT3*^*Cre*,^^10^) to obtain Tm[TMC2] expression in IHCs only. Confocal imaging confirmed that recombination occurred specifically in IHCs where GFP expression was detected (Supplemental Fig. 1A**)**. ABRs were measured at 4 weeks in *Tm*[*Tmc2*]^*VT3Cre/*+^ and control wild type C57BL6J mice. The data show that ABRs were not altered over the entire frequency range. Slight improvement was noticed at 22.6 kHz (P < 0.01, n = 8; Supplemental Fig. 1B). Similarly, DPOAE thresholds were not affected (Supplemental Fig. 1C). ABR of similar amplitude and latency were observed in control mice and those that over-expressed *Tmc2* (Supplemental Fig. 1D). These results confirm that expression of Tm[TMC2] (along with GFP) in IHCs does not have a detrimental effect at the cellular and systems level. We next assessed the extent of the functional compensation induced by expression of Tm[TMC2] in IHCs in absence of TMC1 (*Tm*[*Tmc2*]^*VT3Cre/*+^*;Tmc1*^*Δ/Δ*^; Supplemental Fig. 1B). Our data show that no or very high ABR thresholds were measured in these lines. No DPOAE were detected (data not shown). Recordings performed in the absence of *Tmc1* therefore demonstrate that over-expression of *Tmc2* in IHCs does not compensate for the absence of *Tmc1* in IHCs and OHCs.

### *Tm*[*Tmc2*] expression compensates for deletion of *Tmc1, Tmc2* in vestibular organs

**Figure 8:**
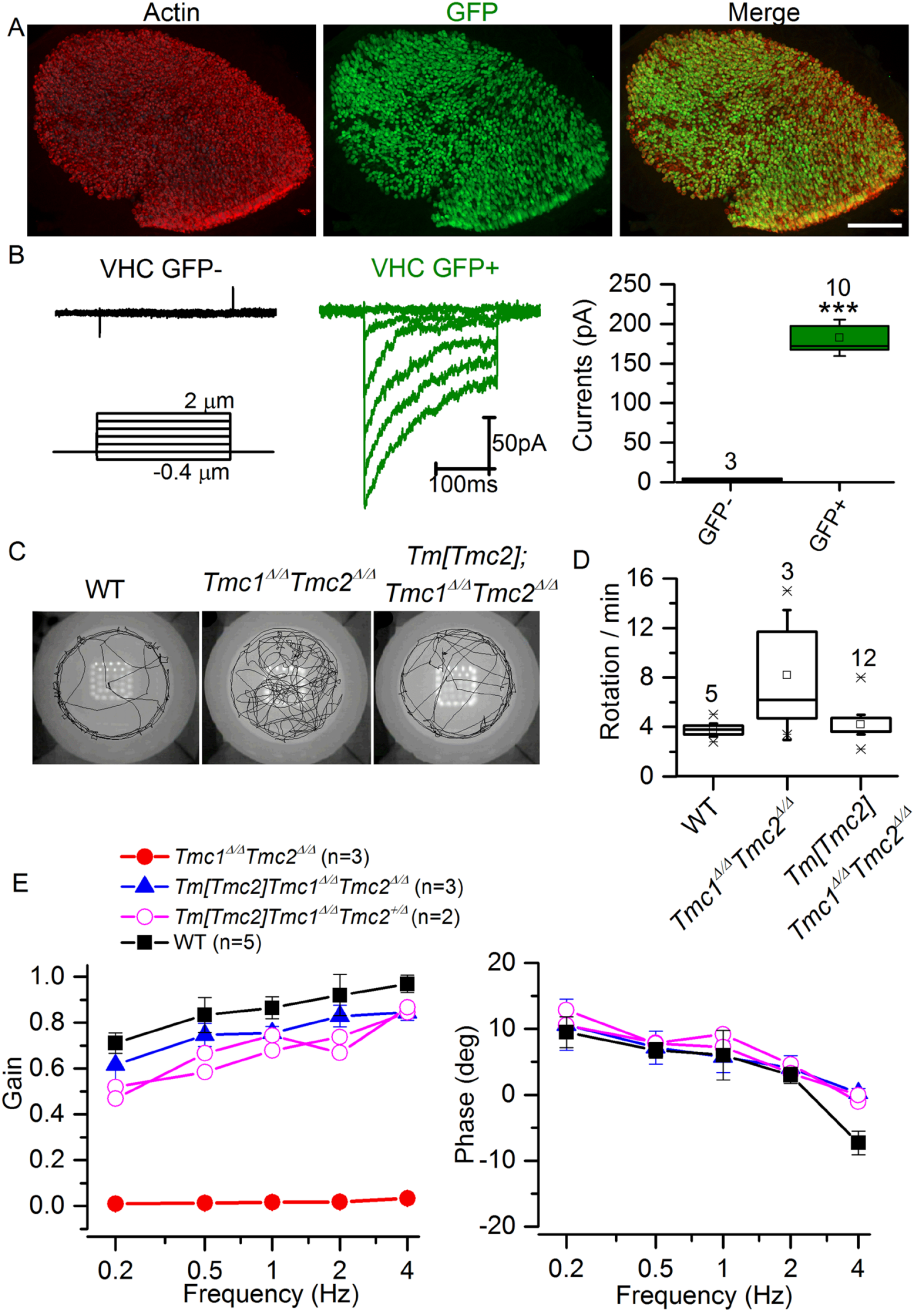
Recovery of sensory transduction and balance function with *Tm*[*Tmc2*]. (**A**) *Gfi1*^*cre*^ recombination in *Rosa26*^*tm*^(*CAG-LSL-Tmc2-IRES-GFP*) leads to GFP expression in hair cells of the utricle, here shown at P8 in *Tmc1*^*Δ/Δ*^*Tmc2*^*Δ/*+^. Red: Actin-phalloidin; Green: GFP. Scale bar: 100 μm. (**B**) Sensory transduction is absent from GFP-negative cells but robust in GFP-positive cells in P3-P6 utricles of *Gfi1*^*cre*^ recombined mice under *Tmc1*^*Δ/Δ*^*Tmc2*^*Δ/Δ*^ background. (**C**) Expression of *Tm*[*Tmc2*] decreases circling behavior that is detected in absence of *Tmc1* and *Tmc2*. Open field observations were performed for 5 min in 4 weeks old wild type C57BL6 (WT), *Tmc1*^*Δ/Δ*^*Tmc2*^*Δ/Δ*^, and *Tmc1*^*Δ/Δ*^*Tmc2*^*Δ/Δ*^ *expressing Tm*[*Tmc2*] after *Gfi1*^*cre*^ recombination. Representative tracks over 2.5 min are shown. While *Tmc1*^*Δ/Δ*^*Tmc2*^*Δ/Δ*^ mice explore the entire field and perform repetitive full body rotations, *Tm*[*Tmc2*]*; Tmc1*^*Δ/Δ*^*Tmc2*^*Δ/Δ*^ mice demonstrate normal behavior similar to wild type controls. (**D**) Rotations were reduced in *Tmc1*^*Δ/Δ*^*Tmc2*^*Δ/Δ*^ expressing *Tm*[*Tmc2*]. The box plot illustrates the mean ± S.D. and median value for the number of rotations covered per minute for each genotype. (**E**) Rotational VOR responses to sinusoidal head rotation were recovered in *Tmc1*^*Δ/Δ*^*Tmc2*^*Δ/Δ*^ expressing *Tm*[*Tmc2*]. Gains and phases are plotted as functions of head rotation frequency for each group. Mean ± S.E.M for groups that have 3 or more mice. Pink lines with hollow circles in panel E represent individual animals due to sample size = 2. ***P < 0.001 (one way ANOVA).

To determine if recombination also occurred in the vestibular organs, we imaged utricles from *Tm*[*Tmc2*]*;Tmc1*^*Δ/Δ*^;*Tmc2*^*Δ/Δ*^ mice. We observed GFP expression in a large number of hair cells at P8 (Fig. 8A) and expression was still prominent by 8 weeks of age (data not shown). *Tmc1*^*Δ/Δ*^*Tmc2*^*Δ/Δ*^ hair cells lack sensory transduction entirely^7^. GFP-negative utricle hair cells of *Tm*[*Tmc2*]*;Tmc1*^*Δ/Δ*^ *Tmc2*^*Δ/Δ*^ are presumed to lack Tm[TMC2] and therefore predicted to also lack sensory transduction. Recording from GFP-negative utricle hair cells of *Tm*[*Tmc2*]*; Tmc1*^*Δ/Δ*^;*Tmc2*^*Δ/Δ*^ indeed lacked transduction entirely (Fig. 8B). On the contrary, expression of Tm[TMC2] in the same mice was associated with large sensory transduction currents in GFP-positive utricle hair cells at P3-P6 (182.5 ± 48.3pA, n = 10; Fig. 8B). These currents were similar to those recorded from utricle hair cells of wild-type neonatal mice^7^. *Tmc1*^*Δ/Δ*^*Tmc2*^*Δ/Δ*^ display severe balance deficits with overtly abnormal vestibular function that include head-bobbing and circling behavior^7^. To assess if *Tm*[*Tmc2*] can compensate for absence of endogenous *Tmc1* and *Tmc2*, we assessed open field behavior in *Tmc1*^*Δ/Δ*^*Tmc2*^*Δ/Δ*^ mice and *Tm*[*Tmc2*]*;Tmc1*^*Δ/Δ*^;*Tmc2*^*Δ/Δ*^. At 4 weeks of age, reduced circling behavior was observed in *Tm*[*Tmc2*]*;Tmc1*^*Δ/Δ*^;*Tmc2*^*Δ/Δ*^ mice (Fig. 8C,D). Rotational vestibulo-ocular reflexes (RVOR) were recorded from wild type C57BL6 (WT), *Tmc1*^*Δ/Δ*^*Tmc2*^*Δ/Δ*^, *Tm*[*Tmc2*]*;Tmc1*^*Δ/Δ*^;*Tmc2*^*Δ/Δ*^ and *Tm*[*Tmc2*]*;Tmc1*^*Δ/Δ*^*;Tmc2*^+*/Δ*^ mice. Data shown in Fig. 8E summarize the gains and phases of the RVOR responses for the four groups of mice. While the WT group exhibited high gain compensatory responses at all the frequencies, *Tmc1*^*Δ/Δ*^*Tmc2*^*Δ/Δ*^ mice exhibited virtually zero gain responses at the same frequencies. However, expression of *Tm*[*Tmc2*] in *Tmc1*^*Δ/Δ*^*Tmc2*^*Δ/Δ*^ lead to robust responses at these frequencies. The phases were nearly identical to those of the WT group. The data demonstrate that *Tm*[*Tmc2*] expression can compensate functionally for loss of endogenous *Tmc1* and *Tmc2* expression in the vestibular system. This compensation is evident at the cellular level in neonatal mice and at the behavioral level in adult mice.

## Discussion

Are TMC1 and TMC2 complementary, redundant, or neither? Genetic redundancy associated with functional redundancy has led to generation of double or triple knockout models for numerous studies^14–16^. Both TMC1 and TMC2 are expressed in neonatal hair cells^6–8^ and may have arisen from gene duplication. Of the two, we suspect that TMC2 may be primordial as it appears to play a more prominent role in the more early evolved vestibular organs. However, either protein can enable sensory transduction in auditory hair cells when expressed in *Tmc1*^*Δ/Δ*^*Tmc2*^*Δ/Δ*^ mice^7,17^. Whether they function as linkers, chaperones or channels, our data support the hypothesis that they perform similar and somewhat redundant functions.

In the cochlea of control mice, endogenous *Tmc2* gene expression declines dramatically over the first postnatal week, while *Tmc1* expression rises and is maintained into adulthood^7^. Previous work has demonstrated that while hair cells that express either *Tmc1* or *Tmc2* maintain mechanosensory function, they possess distinct biophysical properties^8,18^. In particular, different unitary currents, calcium permeability and affinity for dihydrostreptomycin (DHS) are associated with *Tmc1* and *Tmc2* expression^8,18,19^. Hair cells that express *Tmc2* possess larger single-channel conductance with higher calcium permeability and reduced affinity for DHS relative to *Tmc1*-expressing cells^8,18,19^. Although these differences are measureable, prior to this study, it was unclear whether the differences are consequential for normal auditory function. We report here that Tm[TMC2] protein can compensate for TMC1 at the cellular level but not at the systems level for auditory function.

While compensation for TMC1 by TMC2, and vice-versa, has been demonstrated *in vitro*, we now show that Tm[TMC2] can partially compensate for TMC1 *in vivo*. Expression of Tm[TMC2] in neonatal mice led to early acquisition of sensory transduction, longer preservation of transduction, and increased hair cell survival (Supplemental Fig. 2) in the absence of TMC1. Cellular compensation led to partial functional restoration at the systems level: while *Tmc1*^*Δ/Δ*^*Tmc2*^*Δ/Δ*^ are fully deaf, expression of Tm[TMC2] in *Tmc1*^*Δ/Δ*^ or *Tmc1*^*Δ/Δ*^*Tmc2*^*Δ/Δ*^ mice led to ABR recovery for high sound intensity in P16 mice. However, the ABR response disappeared by one month of age, suggesting the recovery was transient, despite persistent expression of Tm[TMC2]. Comparable thresholds have previously been reported in *Tmc1*^*Δ/Δ*^ mice injected with *AAV1.Tmc2*^17^. However, in our experiments, the partial functional compensation at P16 and lack of compensation at later stages may have arisen from incomplete Cre-recombination. If too few hair cells expressed Tm[TMC2], recovery of auditory function may have been limited. Mosaicism of Cre-recombination was evident in OHCs of *Gfi1*^*Cre*^-expressing mice (Fig. 1). While Yang *et al*.^9^ showed strong recombination efficiency (>93%) in the cochlea of *Gfi1*^*Cre*^ mice using a *Rosa26*^*LacZ*^-reporter line, our data suggest a lower recombination rate in *Tm*[*Tmc2*] mice, particularly in OHCs. Although *Gfi1*^*cre*^ mice have been shown to suffer from high frequency hearing loss^13^, no hair cell loss was observed in this strain at 6 weeks of age (Supplemental Fig. 2). Alternatively, or perhaps in addition, the low expression rate of Tm[TMC2] in OHCs may be due to variations in CAG promoter activity in different hair cells^20^. In either case, mosaic expression of *Tm*[*Tmc2*] in OHCs of *Tmc1*^*Δ/Δ*^ mice may have led to insufficient numbers of functional OHCs along the cochlea. However, in the companion paper, Nakanishi *et al*.^21^ used an alternate approach to drive exogenous expression of *Tmc2* in all hair cells and found a similar lack of auditory function, suggesting that the number of hair cells expressing Tm[TMC2] protein is not the primary cause of the inability of *Tm*[*Tmc2*] to compensate for *Tmc1*. Rather, we suggest there is a fundamental difference between the function of TMC2 and TMC1 and that TMC1 expression is essential for normal auditory function.

Interestingly, we found that *Tm*[*Tmc2*] expression does compensate for loss of endogenous *Tmc1* and *Tmc2* in vestibular organs. *Tm*[*Tmc2*]*;Tmc1*^*Δ/Δ*^*;Tmc2*^*Δ/Δ*^ mice had hair cells with normal sensory transduction currents. In contrary to their *Tmc1*^*Δ/Δ*^*;Tmc2*^*Δ/Δ*^ counterpart, *Tm*[*Tmc2*]*;Tmc1*^*Δ/Δ*^*;Tmc2*^*Δ/Δ*^ mice had normal VOR and open field behavior demonstrating normal balance function. As such, we suggest that *Tm*[*Tmc2*] is sufficient for normal vestibular but not auditory function. It is known that, in the vestibular system, *Tmc2* expression is maintained in mature hair cells^7^. Our current findings reinforce the role of TMC2 in the vestibular organs. But why would a protein that mediates mechanosensory transduction operate in vestibular organs but not auditory organs? There are several prominent differences between auditory and vestibular organs, and perhaps there are differences between TMC1 and TMC2 function that make them more suitable for auditory and vestibular function, respectively.

First, an endocochlear potential (EP) of +110 to +125 mV, which develops during the second and third postnatal week in mice^22^, is present in the auditory organ, but not in vestibular organs^23,24^. The EP provides a strong driving force that dramatically increase ionic flux into hair cells^25^. At the same time, calcium concentration near the OHC stereocilia is buffered by the tectorial membrane^22^ which leads to an increase of the channel open probability at rest, thereby enabling them to operate at maximum sensitivity at threshold^26^. The open probability is dramatically decreased when the tectorial membrane is absent^27^. Because cells that express TMC2 have larger single-channel conductance^8^, higher calcium permeability^8,18^ and slower adaptation rates^8^, it is possible that TMC2 expression instead of TMC1 in the cochlea leads to excessive depolarization, subsequent calcium and potassium overload and hair cell toxicity^18^. Since the EP is highest in the basal turn of the cochlea^28^, hair cell death may be more prominent in this region of the cochlea. Our data support this hypothesis, as hair cell survival is noticeably greater in the apical turn in *Tm*[*Tmc2*];*Tmc1*^*Δ/Δ*^ mice (Supplemental Fig. 2).

Second, the cochlea is sensitive to high frequencies, whereas the vestibular organs are sensitive to low frequencies. Perhaps TMC1 includes evolutionary adaptations that enable sensitivity to high frequency stimulation, while TMC2 is needed for sensitivity to low frequencies. Cochlear amplification enhances frequency tuning and sensitivity of the mammalian cochlea at high frequencies that can extend to over 100 kHz^29–32^. It is tempting to speculate that TMC1 provides a function distinct from TMC2 that is necessary for normal OHC function and cochlear amplification. It is possible that normal OHC function depends upon the distinct biophysical properties of TMC1 and the unique homeostatic environment of the cochlea to achieve maximum efficiency of the electromechanical feedback that drives amplification^26^.

Alternatively, mature cochlear hair cells may lack the appropriate molecular partners to bind TMC2 and therefore unable to fully utilize Tm[TMC2] for normal auditory function. For example, *Mus musculus* TMC1 and TMC2 share 57% identity with 72% homology. Their intracellular N-terminal regions, comprised of >50% charged amino-acids, may play an important role for assembly of TMC proteins with interacting partners. Differences in the N-terminal region of the two TMC proteins may account for functional differences.

Giese *et al*.^33^ recently demonstrated that the calcium-binding protein, CIB2, interacts with TMC1 and is essential for sensory transduction in auditory hair cells. The CIB2 interaction with TMC1 is mediated by a region of 50 amino acids in the N-terminal region. This region has 52% identity and 76% similarity with TMC2 which is, perhaps, different enough to lead to different levels of interaction with CIB2. If interaction with CIB2 is required for normal sensory transduction, the amino acid differences in the N-terminal region of TMC2 may account for reduced CIB2 interaction and hence the loss sensory transduction. Interestingly, deletion of CIB2 disrupts auditory function but not vestibular function, suggesting that TMC2 expression and normal vestibular hair cell function do not require CIB2. PCDH15, which forms the lower tip link, has also been shown to bind to TMC1 and TMC2^34,35^. Perhaps different interactions with PCDH15 and other, yet to be identified, molecular partners contribute to the differences between TMC1 and TMC2 function in auditory and vestibular hair cells.

## Methods

### Generation of the *Rosa26*^tm^*(CAG-LSL-Tmc2-IRES-GFP) mouse model*

The targeting vector was constructed by cloning the cDNA of mouse *Tmc2* into the CAG-STOP-GFP-ROSA targeting vector CTV (a gift from Klaus Rajewsky; Addgene plasmid # 15912) between *LoxP-Stop-LoxP* (LSL) cassette and IRES-GFP by the restriction enzyme *Asc*I. The *SgfI* linearized construct was electroporated into C57BL/6 embryonic stem (ES) cells. Neomycin resistant ES cell colonies were screened by PCR to identify those that had undergone homologous recombination. Their karyotype was analyzed to identify clones that had the best chance for germline transmission. Correctly targeted ES cells were expanded and then injected into albino C57BL/6 J blastocysts. Chimeric founders were bred with C57BL/6 J to generate germline heterozygotes. The knockin and *Cre* expressing lines were crossed to previously described^7^ *Tmc1*^*Δ/Δ*^ and *Tmc2*^*Δ/Δ*^ mice to obtain *Tm*[*Tmc2*] transgene expression in mice lacking *Tmc1*, *Tmc2* or both.

### Genotyping

Genotyping for *Gfi1*^*Cre*^ was done using the following primer set: Gfi1CreR: GCCCAAATGTTGCTGGATAGT; Gfi1F: GGGATAACGGACCAGTTG and Gfi1R: CCGAGGGGCGTTAGGATA. Band size was 600 bp for wild type and 700 bp for Cre positive mice. Genotyping *for Rosa26*^*tm*^(*CAG-LSL-Tmc2-IRES-GFP*) was performed with three primers: genoRosa5′.F: AAAGTCGCTCTGAGTTGTTATCAGT; genoRosa3′.RV: GTCTAACTCGCGACACTGTAATTT; LoxPStop.RV: CTATGAACTAATGACCCCGTAATTG, leading to bands size of 539 bp (wild type) and 387 bp for *Rosa26*^*tm*^(*CAG-LSL-Tmc2-IRES-GFP*) knockin mice.

### Quantitative-PCR

To assess expression of *Tm*[*Tmc2*], we performed quantitative PCR using a primer pair targeting *GFP*. Cochlea were rapidly harvested and fast frozen in liquid nitrogen. Total RNA was prepared with Quick-RNA microPrep kit (Zymo research) from single cochlea harvested from *Rosa26*^*tm*^(*CAG-LSL-Tmc2-IRES-GFP*) mice under *Tmc1*^*Δ/Δ*^ *Tmc2*^*Δ/*+^ background crossed with Gfi1^cre/+^ mice at P1, P8 and P20. 100 ng of total RNA per sample was used for reverse transcription using iScript advanced cDNA synthesis kit for RT-qPCR (Biorad). cDNA generated from 5 ng of total RNA was used for a single quantitative PCR reaction. The gene expression levels of β*-actin* and *GFP* were analyzed by single color real-time PCR using CFX Connect Real-Time PCR detection system (Biorad) and SYBR GreenER qPCR supermix (Invitrogen) and calculated with the comparative ∆Ct method as described previously^36^. Each Q-PCR reaction was done in triplicates. The cycle threshold (Ct) for each sample was obtained by averaging triplicates and normalizing relative to the Ct value of β-*actin* from the same cDNA sample. The fold difference in expression for each sample was calculated by normalization to the P1 *Cre*-neg control. Statistical analysis were performed with a minimum of three biological replicates. Primer sequences used for Q-PCR: β-actin Forward: TGAGCGCAAGTACTCTGTGTGGAT; β-actin Reverse: ACTCATCGTACTCCTGCTTGCTGA; GFP Forward: CCACATGAAGCAGCACGACTTCT; GFP Reverse: TGTAGTTGCCGTCGTCCTTGAAGA.

### Immunostaining

Temporal bones were removed after euthanasia in 4 to 8 weeks old mice and placed in 4% PFA for 1 hour, followed by decalcification for 24 to 36 hours with 120 mM EDTA. The sensory epithelium was then dissected out, permeabilized with 0.01% Triton-X and incubated for 24–48 hours with primary antibodies. Rabbit anti-MYO7A primary antibody (1:500, #25–6790, Proteus Bioscience, CA) and Goat anti-GFP (1:500, #NB100–1770, Novus) were applied for 48 hours. Secondary antibodies, Alexa-488 anti-goat and Alexa-633 anti-rabbit IgG (1:200, Invitrogen) were applied for 2–3 hours along with Alexa phalloidin 633 to label actin filaments (Invitrogen, 1:200). Images were obtained on a LSM710 and LSM800 Zeiss confocal microscope (IDDRC Imaging Core grant P30 HD18655) and processed with Zeiss LSM image viewer 4.2.

### Tissue preparation and electrophysiology

Cochlea and utricles were prepared as described previously^8^. Recordings were performed in acutely dissected tissues up to P7 or tissues maintained in culture from P6-P7 up to 30 days (equivalent to P37) in standard MEM (Invitrogen) supplemented with 10 mM HEPES, 0.05 mg/ml ampicillin, 0.05 mg/ml ciprofloxacin and 1.5% fetal bovine serum (Life technologies). Electrophysiological recordings were performed in standard artificial perilymph solution containing (in mM): 140 NaCl, 0.7 NaH_2_PO_4_, 5.8 KCl, 1.3 CaCl_2_, 0.9 MgCl_2_, 5.6 D-glucose, and 10 HEPES-NaOH, adjusted to pH 7.4 and 310 mOsmol/kg. Vitamins (1:50) and amino acids (1:100) were added from concentrates (Invitrogen, Carlsbad, CA). Hair cells were viewed from the apical surface using an Examiner-A1 microscope (Zeiss) equipped with a 63 × water immersion objective with differential interference contrast optics. Recording pipettes (2–4 MΩ) were pulled from R6 capillary glass (King Precision Glass). Mechanotransduction currents were recorded under whole-cell voltage-clamp at a holding potential of −84mV at room temperature (22–24 °C) with an intracellular solution containing (in mM): 135 CsCl, 5 EGTA-CsOH, 10 HEPES, 2.5 Na_2_ATP, 3.5 MgCl_2_, 0.1 CaCl_2_, pH 7.4 and 285 mOsmol/kg. Data were acquired using the Axopatch 200B amplifier (Molecular devices) or the Multiclamp 700 A amplifier (Molecular Devices). Signal was filtered at 10 kHz with a low pass Bessel filter, digitized at ≥20 kHz with a 12-bit acquisition board (Digidata 1440 A, Molecular Devices) and acquired with pClamp 10.5 (Molecular Devices). Electrophysiology data were analyzed offline with OriginLab 2016 software (OriginLab Corporation) and are presented as means ± standard deviation unless otherwise noted.

### Mechanical Stimulation

For OHCs and IHCs recordings, angled stiff glass probes were fabricated from capillary glass using a fire polisher (MF-200, WPI) to create a rounded probe tip of ~3–5 µm in diameter. Mechanical step stimuli were transmitted to the stereocilia bundle as previously described^11^. Briefly, the back end of the glass probe was mounted on a one-dimensional PICMA chip piezo actuator (Physik Instrumente) and driven by a LVPZT amplifier (E-500.00, Physik Instrumente). Voltage steps were used to evoke bundle deflections with a stimulus filtered at 10 kHz by a low-pass 8-pole Bessel filter (Krohn-Hite) to eliminate residual probe resonance. For vestibular hair cells, a pipette filled with standard extracellular solution and a tip diameter of ~ 600 nm was approached and coupled to the kinocilium by gentle suction. Deflections were evoked by applying voltage steps to the piezoelectrical device which consisted of two bimorphs mounted in series and directly coupled to the stimulus probe.

### Auditory Brainstem Responses (ABRs) and Distortion Product Otoacoustic Emissions (DPOAEs)

ABR and DPOAE were recorded from mice anesthetized with xylazine (5–10 mg/kg i.p.) and ketamine (60–100 mg/kg i.p.). Recordings were performed as described previously^37^. For ABR recordings, the ear canal was presented with 5-millisec tone pips. The responses were amplified (10,000 times), filtered (0.1–3 kHz), and averaged with an analog-to-digital board in a PC based data-acquisition system (EPL, Cochlear function test suite, MEEI, Boston). Sound level was raised in 5 to 10 dB steps from 0 to 110 dB sound pressure level (decibels SPL). At each level, 512 responses were averaged (with stimulus polarity alternated) after “artifact rejection”. Threshold was determined by visual inspection. Data were analyzed and plotted using Origin-2016 (OriginLab Corporation). Thresholds averages ± standard deviations are presented unless otherwise stated. For DPOAE, f1 and f2 primary tones (f2/f1 = 1.2) were presented with f2 varied between 5.6 and 45.2 kHz in half-octave steps and L1–L2 = 10 dB SPL. At each f2, L2 was varied between 10 and 80 dB SPL in 10 dB SPL increments. DPOAE threshold was defined from the average spectra as the L2-level eliciting a DPOAE of magnitude 5 dB SPL above the noise floor. The mean noise floor level was under 0 dB across all frequencies. Stimuli were generated with 24-bit digital I–O cards (National Instruments PXI-4461) in a PXI-1042Q chassis, amplified by an SA-1 speaker driver (Tucker–Davis Technologies, Inc.), and delivered from two electrostatic drivers (CUI CDMG15008-03A) in our custom acoustic system.

### Open field

The open field test was conducted using a circular frame measuring 42 cm in diameter, placed inside a sound chamber with overhead LED lighting, set to 30 lux at the center, inside a dimmed room. Mice were placed one at a time inside the circular open field, and allowed to explore for 5 min. Behavior was recorded and tracked using Ethovision XT, enabling measures of distance traveled and velocity. Open field assessments were all conducted blind.

### Animals and physiological recordings (Brighton, UK)

All mice used for measurements in this study at the University of Brighton were imported from Boston Children’s Hospital. The mice were kept under standard housing conditions with a 12 h/12 h dark-light cycle and with food and water ad libitum. Genotyping was performed in the Holt/Geleoc Lab. All procedures involving animals were performed in accordance with UK Home Office regulations with approval from the University of Brighton Animal Welfare and Ethical Review Body.

Mice, 3–6 weeks of age, were anesthetized with ketamine (0.12 mg/g body weight i.p.) and xylazine (0.01 mg/g body weight i.p.) for nonsurgical procedures or with urethane (ethyl carbamate; 2 mg/g body weight i.p.) for surgical procedures. The animals were tracheotomized, and their core temperature was maintained at 38 °C. To measure BM displacements, cochlear microphonics, a caudal opening was made in the ventro-lateral aspect of the right bulla to reveal the round window. Cochlear microphonic potentials (CM) were measured from the round window membrane by using glass pipettes filled with artificial perilymph, with tip diameters of 50 to 100 μm (recording bandwidth >30 kHz). Signals were amplified with a recording bandwidth of DC − 100 kHz using a laboratory designed and constructed preamplifier. With low impedance electrodes, CM was measured at levels of 20 dB SPL in response to 5 kHz tones in mice with DPOAE responses that were sensitive throughout the 1–70 kHz range of the sound system. Intracellular electrodes (70–100 MΩ, 3 M KCl, filled) were pulled from 1 mm O.D., 0.7 mm I.D quartz glass tubing on a Sutter P-2000 micropipette puller (Sutter Instrument Novato, CA 94949, USA). Signals were amplified and conditioned using laboratory built pre-amplifiers and conditioning amplifiers. Electrodes were advanced using a piezo activated micropositioner (Marzhause GMBH). The pipette tip was inserted through the round window membrane and into the BM, close to the feet of the OPCs, under visual control. The first cells to be encountered had resting potentials ≤−80 mV, could be held for 10 s of minutes and were assumed to be supporting cells. Other cells encountered immediately before penetrating the scala media had resting potentials of ~ −50 mV and could be held for seconds to several minutes. These were presumed OHCs. Loss in sensitivity of the preparation was determined by changes in CM threshold. Losses were never encountered as a consequence of intracellular penetration with the electrode. Experiments were terminated immediately there was any loss in CM threshold (≥5 dB SPL) due usually to change in the condition of the preparation.

Sound was delivered via a probe with its tip within 1 mm of the tympanic membrane and coupled to a closed acoustic system comprising two MicroTechGefell GmbH 1-inch MK102 microphones for delivering tones and a 0.25-inch microphone for monitoring sound pressure at the tympanum. The sound system was calibrated *in situ* for frequencies between 1 and 70 kHz by using a laboratory designed and constructed measuring amplifier, and known sound pressure levels (SPLs) were expressed in dB SPL with reference to 2 × 10^−5^ Pa. Tone pulses with rise/fall times of 1 ms were synthesized by a Data Translation 3010 (Data Translation, Marlboro, MA) data acquisition board, attenuated, and used for sound-system calibration and the measurement of electrical and acoustical cochlear responses. To measure distortion product otoacoustic emissions (DPOAEs), primary tones were set to generate 2f1−f2 distortion products at frequencies between 1 and 50 kHz. DPOAE were measured for levels of f1 ranging from 10 to 80 dB SPL, with the levels of the f2 tone set 10 dB SPL below that of the f1 tone. DPOAE threshold curves were constructed from measurements of the level of the f2 tone that produced a 2f1− f2 DPOAE with a level of 0 dB SPL where the frequency ratio of f2:f1 was 1.23. System distortion during DPOAE measurements was 80 dB below the primary tone levels. Tone-evoked BM displacements were measured by focusing the beam of a self-mixing, laser-diode interferometer through the round window membrane to form a 20-μm spot on the center of the basilar membrane in the 50–56 kHz region of the cochlea. The interferometer was calibrated at each measurement location by vibrating the piezo stack on which it was mounted over a known range of displacements. At the beginning of each set of BM measurements it was ensured that the 0.2 nm threshold used as the criterion for threshold was at least as sensitive as the 0 dB SPL threshold for DPOAE before the cochlea was exposed. BM measurements were checked continuously for changes in the sensitivity of the measurement (due to changes in alignment or fluid on the round window) and for changes in the condition of the preparation. If the thresholds of latter changed by more than 5–10 dB SPL, the measurements were terminated. Tone pulses with rise/fall times of 1 ms were used for the basilar membrane measurements. Stimulus delivery to the sound system and interferometer for calibration and processing of signals from the microphone amplifiers, microelectrode recording amplifiers, and interferometer were controlled by a DT3010/32 (Data Translation, Marlboro, MA) board by a PC running Matlab (The MathWorks, Natick, MA) at a sampling rate of 250 kHz. The output signal of the interferometer was processed using a digital phase-locking algorithm, and instantaneous amplitude and phase of the wave were recorded.

All measurements were performed blind. Measurements were made from each animal in a litter and data were analysed at the end of each set of measurements. When all measurements had been made from a particular litter, the tissue was genotyped. Randomization was not appropriate because we had no foreknowledge of the genotype, although we could guess it from the phenotype. Phenotypic differences between the WT, heterozygous and homozygous mice were very strong. Thus only sufficient numbers of measurements were made to obtain statistically significant differences. Experiments were terminated (<5% of all measurements) if the physiological state of the preparation changed during a measurement. Data from such measurements were excluded.

### Eye movement recording and vestibular stimulation (Mississippi Medical Center)

Mice were imported to the University of Mississippi Medical Center from Dr. Geleoc’s laboratory. All procedures were carried out in accordance with NIH guidelines and approved by the Institutional Animal Care and Use Committee at the University of Mississippi Medical Center. All surgical procedures were performed aseptically, as described before^38^. Briefly, each mouse was implanted with a small head holder on the skull and was allowed 7 days to recovery before eye movement tests.

Horizontal and vertical eye position signals were recorded using a video-based eye tracking system (ISCAN ETS-200, ISCAN, Burlington, MA). An infrared camera equipped with a zoom lens (Computar TV Zoom Lens, Computar Optics Group, Japan) was attached to the platform mounted on a servo-controlled rotator/sled (Neurokinetic, Pittsburgh, PA) and was focused on the left eye of each mouse, which was fixed to the platform via the head holder. A multiple infrared LED illuminator attached to the camera produced illumination and a reference corneal reflection (CR) for eye movement measurement. The eye tracker tracked the pupil center and the CR at a speed of 240 frames per second with a spatial resolution of 0.1 deg. Calibration was achieved by rotating the camera from the left 10 degree to the right 10 degree around the vertical axis of the eye. Following the calibration, a series of rotational accelerations were delivered. To measure the steady state VOR responses, horizontal rotations were delivered at 0.2, 0.5, 1, 2 and 4 Hz (60 degree/s peak velocity). Signals related to horizontal and vertical eye position and head position were sampled at 1 kHz at 16 bits resolution by a CED Power 1401 system (Cambridge Electronics Devices, Cambridge, UK). Eye movement responses were analyzed using Spike2 (Cambridge Electronics Devices), MatLab (MathWorks, Natick, MA) and SigmaPlot (Systat Software, San Jose, CA). Eye position signals were filtered and differentiated with a band-pass of DC to 50 Hz to obtain eye velocity signals. As described in details in Stewart *et al*. (2016), gains and phases of the RVORs were calculated by performing Fast Fourier Transform (FFT) on the de-saccaded eye velocity signal and head rotation velocity signal.

### Statistics

Statistical analyses were performed with Origin 2016 (OriginLab Corporation). Data are presented as means ± standard deviations (S.D) or standard error of the mean (S.E.M) as noted in the text and figure legend. One-way analysis of variance (ANOVA) and independent student t-test were used to determine significant differences between the means. In the box plot graphs, the ends of whisker are defined by S.E.M. Central rectangle spans from first quartile to third quartile. The segment in the rectangle indicates median and the square dot indicates the mean.

## Electronic supplementary material

Supplemental figures 
