# Supplementary material for: Transgenic Tmc2 expression preserves inner ear hair cells and vestibular function in mice lacking Tmc1
Source: Sci Rep. 2018 Aug 14;8:12124. doi: 10.1038/s41598-018-28958-x (PMC6092434; doi:10.1038/s41598-018-28958-x)
Supplement: Supplementary file 1 — Supplemental figures [file 41598_2018_28958_MOESM1_ESM.pdf]

SUPPLEMENTAL INFORMATION

Transgenic *Tmc2* expression preserves inner ear hair cells and vestibular function in mice lacking *Tmc1*

Yukako Asai[1]*, Bifeng Pan*[1]*, Carl Nist-Lund*[1]*, Alice Galvin*[1]*, Andrei N. Lukashkin*[2]*, Victoria A. Lukashkina*[2]*, Tianwen Chen*[3]*, Wu Zhou*[3]*, Hong Zhu*[3]*, Ian J. Russell*[2]*, Jeffrey R. Holt*[1,4] *& Gwenaelle S. G. Géléoc*[1]

[1]Dept. of Otolaryngology, F.M. Kirby Center for Neurobiology; Boston Children’s Hospital, Harvard Medical School, Boston, MA.

[2]Sensory Neuroscience Research Group, School of Pharmacy and Biomolecular Sciences, University of Brighton, Brighton, UK.

[3]Department of Otolaryngology and Communicative Sciences, University of Mississippi Medical Center.

[4]Dept. of Neurology, Boston Children’s Hospital, Harvard Medical School, Boston, MA.

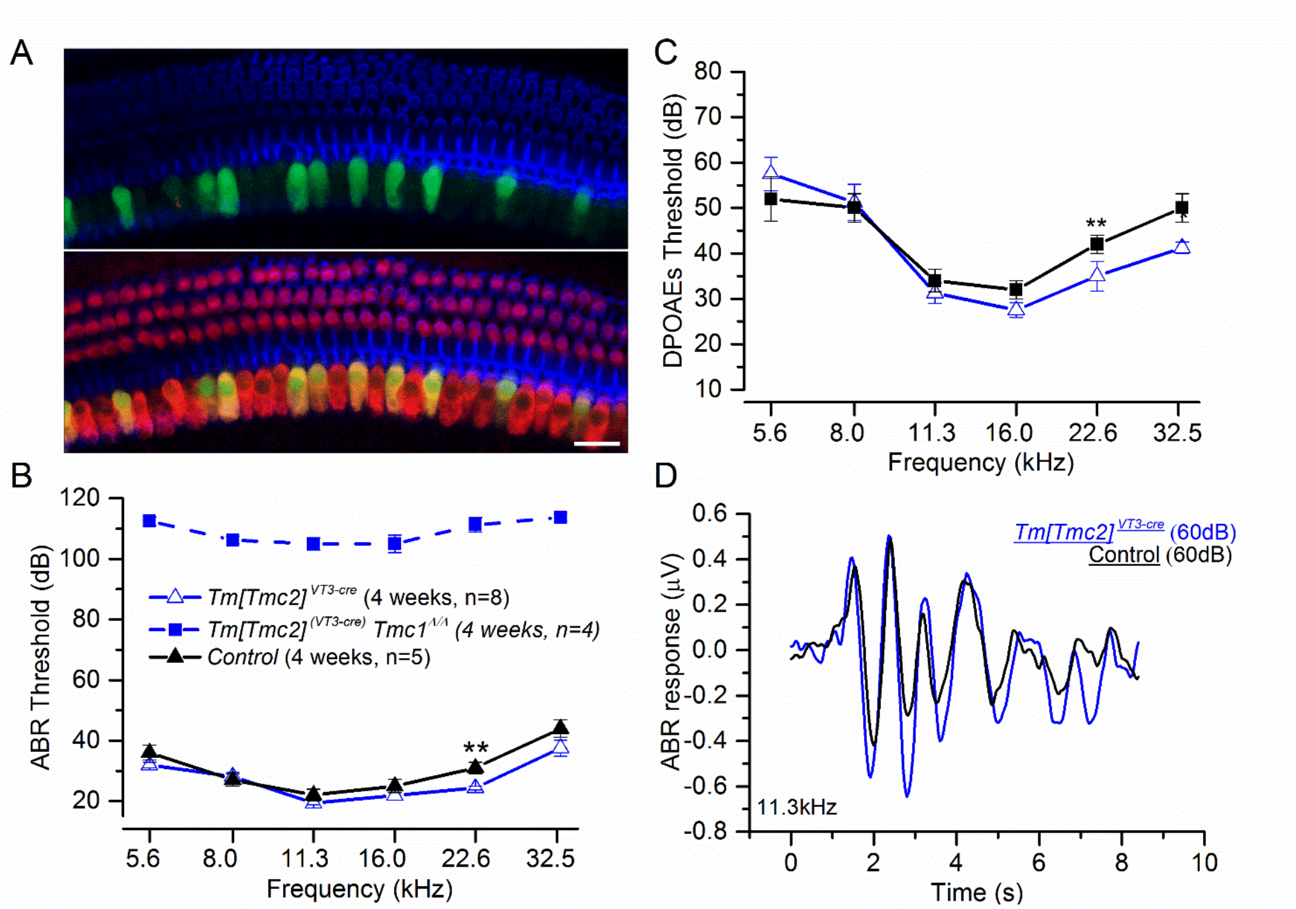

Supplemental Figure 1: *VT3*^{Cre} inducible expression of *Tm[Tmc2]* in IHCs does not alter auditory thresholds in control mice but fails to restore ABR sensitivity in absence of *Tmc1*. A- *VT3*^{Cre} excision of the stop codon leads to GFP expression in IHCs exclusively. Expression was visible at P16 in some but not all IHCs. Hair cells are labeled with rabbit anti-MYOVIIa (red), GFP signal is amplified with a goat anti-GFP antibody (green) and phalloidin counterstain labels actin filaments (blue). Apical turn, scale bar 20µm. B- ABR were similar in C57BL6 wild type control and *Tm[Tmc2];Tmc1*$^{+/+}$ mice but elevated thresholds were observed in *Tm[Tmc2];Tmc1*$^{\Delta/\Delta}$ mice. C- Similarly DPOAE were absent in in *Tm[Tmc2];Tmc1*$^{\Delta/\Delta}$ but unaffected in *Tm[Tmc2];Tmc1*$^{+/+}$ mice. D- Increase in peak 1 amplitude was observed in *Tm[Tmc2];Tmc1*$^{+/+}$ suggesting a slight increase in sensitivity resulted from expression of the transgene. ABR response shown for 60dB exposure of an 11.3 kHz pure tone. Recordings were all performed in 4 weeks old mice. ** $P<0.01$

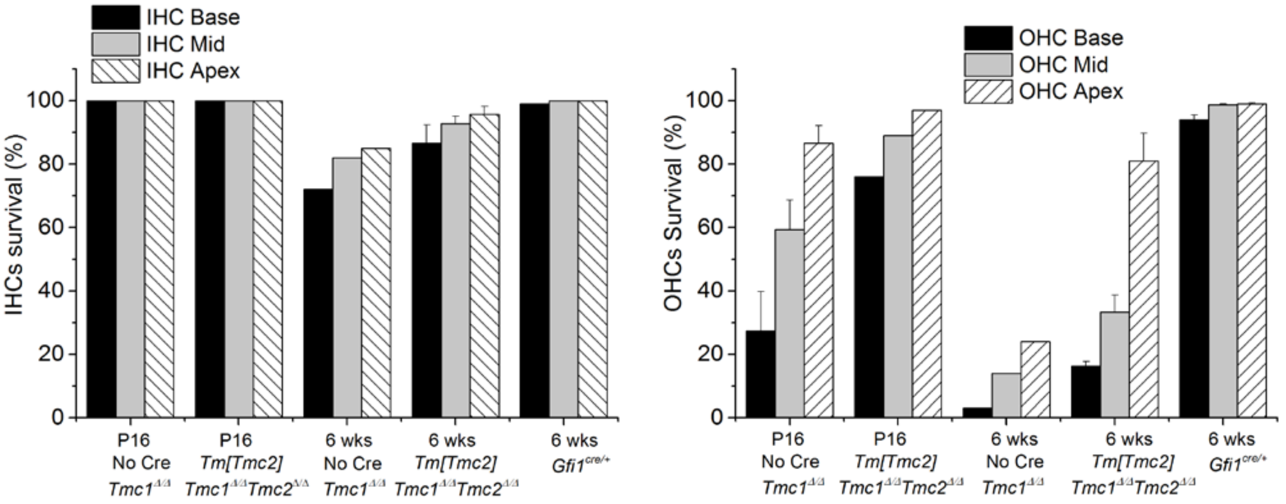

<u>Supplemental Figure 2</u>: Hair cell survival in presence or absence of the transgene at P16 and 6 weeks. To determine if expression of *Tm[Tmc2]* compensates for the loss of endogenous *Tmc2* in Tmc1 null mice, we performed hair cell count in $Tmc1^{\Delta/\Delta}Tmc2^{+/+}$ and $Tmc1^{\Delta/\Delta}Tmc2^{\Delta/\Delta}$ mice expressing *Tm[Tmc2]*. Hair cell counts were performed on fixed inner ears labeled with rabbit anti-MYOVIIA and phalloidin counterstain. Percentage of OHCs were determined from cell counts assuming a ratio of 3.4 times the total number of IHCs (including absent IHCs when that was the case). Sections of similar length were observed in the basal, mid and apical turns.

Data show that IHCs survival did not depend upon the presence of *Tm[Tmc2]* up to 6 weeks. However OHCs survival was much greater in *Tm[Tmc2]* mice at P16 in comparison with those that did not express the transgene. By 6 weeks, severe OHC loss was evident in the basal and mid region of the organ despite presence of *Tm[Tmc2]*. No hair cell loss was observed in $Gfi1^{cre}$ mice up to 6 weeks of age despite increase in high frequency ABR thresholds (Fig 6b) and evidence of age related hearing loss in this strain (Matern et al. 2017). Mean +/- SEM.

Reference:

Matern, M. et al. Gfi1Cre mice have early onset progressive hearing loss and induce recombination in numerous inner ear non-hair cells. Sci Rep 7, 42079, doi:10.1038/srep42079 (2017).
